# Dermotropic *Leishmania donovani* in Sri Lanka: visceralizing potential in clinical and preclinical studies

**DOI:** 10.1017/S003118201700169X

**Published:** 2017-11-08

**Authors:** K. K. G. D. U. L. KARIYAWASAM, A. SELVAPANDIYAN, H. V. Y. D. SIRIWARDANA, A. DUBE, P. KARUNANAYAKE, S. A. S. C. SENANAYAKE, R. DEY, S. GANNAVARAM, H. L. NAKHASI, N. D. KARUNAWEERA

**Affiliations:** 1 Department of Parasitology, Faculty of Medicine, University of Colombo, No. 25, Kynsey Road, Colombo 8, Sri Lanka; 2 JH-Institute of Molecular Medicine, Jamia Hamdard, New Delhi, India; 3 Central Drug Research Institute, Sector 10, Jankipuram Extension, Sitapur Road, Lucknow, Uttar Pradesh 226031, India; 4 Department of Clinical Medicine, Faculty of Medicine, University of Colombo, No. 25, Kynsey Road, Colombo 8, Sri Lanka; 5 Laboratory of Emerging Pathogens, Division of Emerging and Transfusion Transmitted Diseases, Center for Biologics Evaluation and Research, Food and Drug Administration, Silver Spring, MD, USA

**Keywords:** Leishmaniasis, skin lesions, animal models, cytokines, patient follow-up, virulence

## Abstract

The visceralizing potential of apparently dermotropic *Leishmania donovani* in Sri Lanka (*L. donovani*-SL) was investigated through long-term follow-up of cutaneous leishmaniasis (CL) patients and *in vivo* and *in vitro* experimental infection models. CL patients (*n* = 250) treated effectively with intra-lesional antimony therapy were followed-up six monthly for 4 years. There was no clinical evidence of visceralization of infection (VL) during this period. Infection of BALB/c mice with *L. donovani*-SL (test) through intra-dermal route led to the development of cutaneous lesions at the site of inoculation with no signs of systemic dissemination, in contrast to the observations made in animals similarly infected with a visceralizing strain of *L. donovani*-1S (control). Cytokine (IL-10, IFN-γ) release patterns of splenocytes and lymph node cell cultures derived from mice primed with experimental infections (with either test or control parasites) revealed significantly high IFN-γ response associated with test mice with CL, while prominent IL-10 levels were observed in association with control mice with VL. Furthermore, diminished infection efficiency, intracellular growth and survival of *L. donovani*-SL parasites compared with *L. donovani*-1S were evident through *in vitro* macrophage infection experiments. These studies confirm, for the first time, the essential dermotropic nature of *L. donovani*-SL suggesting natural attenuation of virulence of local parasite strains.

## INTRODUCTION

Leishmaniases are a group of vector-borne diseases caused by intracellular protozoa that belong to the genus *Leishmania.* Clinical manifestations of leishmaniasis comprise three distinct forms, viz; cutaneous leishmaniasis (CL), muco-cutaneous leishmaniasis (MCL) and visceral leishmaniasis (VL) (Alvar *et al.* 2012). In Sri Lanka, leishmaniasis is a recently established disease with over 6500 cases of CL reported so far (Sri Lanka Epidemiology Unit Ministry of Health, 2016), with a few autochthonous (and imported) cases of visceral and mucosal cases (Rajapaksa *et al.* 2005; Abeygunasekara *et al.* 2007). The sandfly *Phlebotomus argentipes* var. *glaucus* was identified as the probable vector for leishmaniasis transmission in Sri Lanka (Gajapathy *et al.* 2013; Senanayake *et al.* 2015). CL is the predominant clinical form in Sri Lanka, caused by a genetic variant of *Leishmania donovani*, viz., *L. donovani*-MON-37 (Karunaweera *et al.* 2003), a usually visceralizing parasite elsewhere (Alam *et al.* 2009). Interestingly the same isotype (*L. donovani*-MON-37) has also been isolated from a VL patient in Sri Lanka (Ranasinghe *et al.* 2012). Sporadic cases of CL due to *L. donovani* have also been observed in other endemic regions for VL (Mebrahtu *et al.* 1993; Pratlongl *et al.* 1995; Ben-Ami *et al.* 2002; Gelanew *et al.* 2011).

Intra-species genomic variations might be associated with the cutaneous localization of *L. donovani* in Sri Lanka (Zhang and Matlashewski, 1997, 2001; Karunaweera *et al.* 2003; Siriwardena *et al.* 2007; Bhattarai *et al.* 2010), with copy number variations of the A2 gene implicated (Zhang *et al.* 2014). In addition to the genomic variations, several hosts, vectors and other parasite determinants are believed to influence the clinical outcome of leishmaniasis (McCall *et al.* 2013). Furthermore, host genetic factors responsible for regulation of cytokine production may also determine the disease outcome (Blackwell, 1998; Blackwell *et al.* 2009).

Determinants of disease outcome in leishmaniasis, such as the developmental stage, infective dose, species/strain and route of infection have been extensively studied over several decades using animal models (Constant *et al.* 2000; Sacks and Noben-Trauth, 2002; Baldwin *et al.* 2003; Roberts, 2005; Nieto *et al.* 2011; Mahmoudzadeh-Niknam *et al.* 2013). Studies on BALB/c mice, have been instrumental in gaining valuable insights into the immunobiology of leishmaniasis (Bucheton *et al.* 2002; Sacks and Noben-Trauth, 2002; Mestas and Hughes, 2004). The interest in using Syrian golden hamsters as an animal model to study leishmaniasis, however, is due to its clinicopathological features that resemble the human disease (Wilson *et al.* 2005; Nieto *et al.* 2011; Loria-Cervera and Andrade-Narvaez, 2014).

Studies on the progression of disease pathology, as evident through clinical and haematological investigations, have indicated the visceralizing potential of dermotropic variants of *Leishmania* species, such as *L. donovani* and *L. infantum* (Ben-Ami *et al.* 2002; Gelanew *et al.* 2011; Santos-Oliveira *et al.* 2011). A study done in the late 1990s on CL causing *L. infantum* in the Mediterranean basin, has established their essential dermotropic nature with no evidence of lymphatic spread or visceral involvement during the follow-up period (Giudice *et al.* 1998). However, there are studies that provide compelling evidence for visceralization following initial cutaneous disease due to *L. donovani* (Ben-Ami *et al.* 2002; Santos-Oliveira *et al.* 2011; Philips *et al.* 2014). Interestingly, the existing literature also suggests that cutaneous lesions may occur either before or after a visceral infection (Giudice *et al.* 1998; Gelanew *et al.* 2011; Santos-Oliveira *et al.* 2011; Philips *et al.* 2014). Therefore, a given parasite species may not always lead to a predictable clinical outcome in terms of host tissue involvement and pathology.

Natural sequelae of infection in CL patients in Sri Lanka are not yet known. Similarly, the molecular and immunological bases of monocyte invasion, intra-cellular multiplication with or without systemic invasion of *L. donovani* in Sri Lanka (*L. donovani*-SL) remain poorly understood. This paper presents the results of the first study, aimed at delineating the visceralizing potential of *L. donovani*-SL through long-term follow-up of CL patients and the use of both *in vivo* and *in vitro* models to study parasite virulence.

## MATERIALS AND METHODS

### Sample collection

Patients (*n* = 250) with skin lesions were identified through passive detection methods, from those who attended dermatology clinics at National Hospital of Sri Lanka, Colombo North Teaching Hospital, Anuradhapura Teaching Hospital and Hambantota General Hospital. Inclusion criteria for the study were patients with cutaneous lesions (papules/nodules/ulcers/plaques) due to leishmaniasis, as confirmed by direct microscopy and/or culture of tissue samples. Patients were recruited for the study to represent all administrative provinces of the country, following informed consent. All patients were subjected to general physical examination by a Physician to exclude any clinical sign(s) of lymphatic or visceral spread and 3 mL sample of blood was taken for investigations at the time of recruitment. The age range of patients recruited for the study was between 11 months and 70 years (median = 40 years) with a male-to-female ratio of 2·5 : 1 (male = 179, female = 71). Exclusion criteria were co-morbidities that cause irregular chronic fever, such as lymphomas and tuberculosis and those on immune-suppressive drugs that make the follow-up procedures complicated.

### Patient diagnosis and treatment

Tissue fluid aspirates and slit-skin scrapings obtained from skin lesions were used to make smears on glass slides and also to inoculate cultures. Giemsa-stained smears were microscopically examined under oil immersion (1000× magnification) (Ihalamulla *et al.* 2002). Polymerase chain reaction (PCR) was done for a subset of samples using a standard protocol (Lachaud *et al.* 2000, 2001). Serum samples from all patients were tested for rk39 antibodies using the dipstick assay according to manufacturer's instructions (InBios, USA). All diagnosed patients were referred to the local dermatologist for treatment. Patients were treated with weekly intra-lesional injections of sodium stibogluconate (100 mg mL^−1^) until cure (IL-SSG). Cure of lesion(s) was defined as the reduction of lesion size (ulceration area in case of ulcers or the induration area in case of non-ulcerative lesions) to zero or flattened as the case may be, as judged by the collaborating dermatologist (Sri Lanka College of Dermatologists, 2013).

### Follow-up of patients

All patients were followed up at six monthly intervals for a period of 4 years. Patients were subjected to general physical examination by a Physician at each follow-up visit (similar to at the time of recruitment), to detect any persisting, recurring or new skin lesions and likely sign(s) of visceralization, such as anaemia, weight loss or hepatosplenomegaly.

### Haematological investigations

Blood samples (3 mL) were collected from each patient at the time of recruitment for the study and at each follow-up visit through venipuncture and used as follows: 1 mL in to an Ethylenediaminetetraacetic acid (EDTA)-coated tube, for estimation of haemoglobin content, packed cell volume and total cell count; 1 mL to analyse serum albumin and globulin levels using an auto analyser (Roche, Switzerland); 1 mL to a plain microcentrifuge tube for serum separation and assay for anti-*L. donovani* antibodies (rk 39 test, InBios International Inc.).

### Parasites

*Leishmania donovani* parasites isolated from CL patients in Sri Lanka (*L. donovani*-SL) and a known visceralizing strain, *L. donovani* 1S (ATCC strain 30 142: origin-Sudan) were used in animal experiments.

### Parasite cultures for animal experiments

Parasites isolated from skin lesions of six CL patients representing different lesion types (nodule = 1, papule = 1, ulcer = 1, plaques = 1, ulcerating nodules = 2) were propagated in a culture medium containing complete M199 (Gibco, Invitrogen, USA), supplemented with 20% heat-inactivated fetal bovine serum (FBS). Maximum of three to four passages were done until they reached the parasite count of 1 × 10^6^ in 1 mL of culture media. Metacyclic promastigotes were purified by Ficoll density gradient centrifugation and were confirmed morphologically using previously described methods (Sacks and Perkins, 1984; Späth *et al.* 2000). Metacyclic promastigotes (1 × 10^6^/10 *µ*L) from each of the six isolates were used in animal experiments.

### *In vivo* animal inoculation with *Leishmania* parasites

A total of 56 female BALB/c mice (aged 5–6 weeks) and 30 male Syrian golden hamsters (40–50 g weight) were included in the study. Animals were moved into an acclimatization room 1 week prior to the commencement of the experiments. Metacyclic promastigotes (at a dose of 1 × 10^6^/10 *µ*L) that were derived from each of six clinical isolates of *L*. *donovani*-SL were used to inject a total of 36 BALB/c mice intra-dermally (ID) through ear pinna (B-ID-SL) as described previously (Selvapandiyan *et al.* 2009; Dey *et al.* 2014). A positive control group included six BALB/c mice inoculated ID with 1 × 10^6^ metacyclic promastigotes of *L. donovani-*1S parasites (B-Ld1S), according to the previously used methods (Nagill and Kaur, 2010). A negative control group included 14 BALB/c mice similarly treated with normal saline. Five Syrian golden hamsters were also infected using similar dosage and methodology, *via* intra-dermal injection to ear pinna [experimental group *n* = 25 (H-ID-SL), negative control group *n* = 5].

### Disease progression in animals

The animals were observed weekly for symptoms/signs of infection, specifically for skin lesions, abdominal swelling due to organ enlargement, and lethargy. Sizes of lesions (if present) and the weight of both mice and hamsters were recorded on a weekly basis. Mice were euthanized in two batches (*n* = 20) at 5 and at 10 weeks (*n* = 36) after inoculation. For the hamster model, euthanasia was performed at 5 months following inoculation. The organs [spleen, lymph nodes (LNs) and liver] were removed, weighed and post-infection changes, if any were recorded. *Leishmania donovani* burden in cutaneous lesions, splenocytes, hepatic cells and lymphocytes were determined using Giemsa-stained dab smears and expressed in Leishman Donovan units (LDU: the number of amastigotes per 1000 host nuclei, multiplied by the weight of the organ).

### Soluble *Leishmania* antigen (SLA) preparation

SLA was prepared by five sequential freeze–thaw cycles of stationary phase promastigotes of *L. donovani*-SL, grown in liquid culture using similar methods as before (Afrin and Ali, 1997; Afrin *et al.* 2002; Ferraz Coelho *et al.* 2003). Briefly, a pellet of 1 × 10^7^ promastigotes mL^−1^ was washed three or four times, in 5 mL of cold sterile phosphate-buffered saline (PBS). After five sequential freeze–thaw cycles, solutions were cleared by centrifugation at 2500 rpm at 4 °C for 5 min and the protein concentration in the supernatant was determined by the Lowry assay (Lowry *et al.* 1951).

### Preparation of cell suspensions from spleen and LNs for *in vitro* stimulation with parasite antigens

Spleens and LNs removed from 12/36 BALB/c mice, 10 weeks after inoculation of *L. donovani*-SL (*n* = 6/12) and *L. donovani*-1S (*n* = 6/12), were used to extract cells for *in vitro* stimulation. Cells were extracted from freshly removed organs and stimulated with soluble parasite antigens.

Splenocytes and lymphocytes were separated into single-cell suspension by passing through a fine wire mesh. The red blood cells were lysed with ACKlysis buffer. Splenocytes and lymphocytes were then re-suspended in culture at 3 × 10^6^ cells mL^−1^ in complete Dulbecco's modified Eagle's medium (DMEM) supplemented with 20 mm 4-(2-hydroxyethyl)-1-piperazineethanesulfonic acid) HEPES, 10% heat-inactivated FBS, 20 U mL^−1^ penicillin, 20 *µ*g mL^−1^ streptomycin and 50 *µ*m *β*-mercapto-ethanol (Sigma) at 37 °C in 5% CO_2_ using 24-well, flat bottom plates (Nunc, USA). Cells were stimulated with SLA at a concentration of 100 *µ*g well^−1^ and incubated at 37 °C for 72 h, prior to collection of culture supernatants for cytokine assays (Selvapandiyan *et al.* 2009).

### Cytokine assay of *in vitro* stimulated culture supernatants

Supernatants were collected from the cultures of splenocytes and LNs cells of infected mice after 72 h stimulation with 40 *µ*L of parasite antigens (100 *µ*g well^−1^). IFN-γ and IL-10 cytokine concentrations in culture supernatants were determined by sandwich ELISA, according to the manufacturer's instructions (R&D SYSTEMS, Minneapolis, USA).

### *In vitro* infection of human macrophages with *L. donovani* promastigotes

Human mononuclear cells were isolated from peripheral blood of normal healthy donors by density gradient sedimentation in Ficoll-Hypaque (Sigma Chemical Co. St. Louis, MO). Monocytes were separated by adherence to eight-well plates and incubated at 37 °C in 5% CO_2_. These cells were re-suspended (1·8 × 10^5^ cells mL^−1^) in RPMI 1640 medium containing 10% FBS and macrophage colony-stimulating factor (20 ng mL^−1^, ProSpec, Israel). The re-suspended cells (0·5 mL) were plated on eight-chamber Lab-tek tissue-culture slides (Nunc Laboratories). The slides were incubated for 6·5 days to facilitate the differentiation into macrophages. The human macrophages were infected with either *L. donovani-*SL *or L. donovani-*1S stationary-phase promastigotes (10 : 1 parasite to macrophage ratio) and were incubated at 37 °C in 5% CO_2_ using previously established methods (Pearson *et al.* 1981). After 6 h of infection, slides were washed thoroughly to remove excess parasites and the numbers of parasites inside the macrophages were counted at time points of 6, 12, 24, 48 and 72 h. At each time point, the culture medium was gently removed and the remaining slides were air-dried and stained with Giemsa stain. To measure the parasite load in these cultures, a minimum of 300 macrophages were counted. Measures of infection efficiency (% infected cells), intracellular growth (parasites per infected cell) and parasite survival (parasites per 100 cells) were recorded. Experiments were repeated ten times.

### Statistical analysis

The resulting data was stored in a database and analysed using SPSS version 19·0. Pearson's Chi-square (*χ*^2^) test was used to assess the difference between groups for categorical variables. Differences were considered significant at *P* < 0·05.

### Ethical considerations

Written informed consent was obtained from all study participants. Ethical approval for the study was granted by the Ethics Review Committee, Faculty of Medicine, the University of Colombo, Sri Lanka.

## RESULTS

**Fig. 1.**
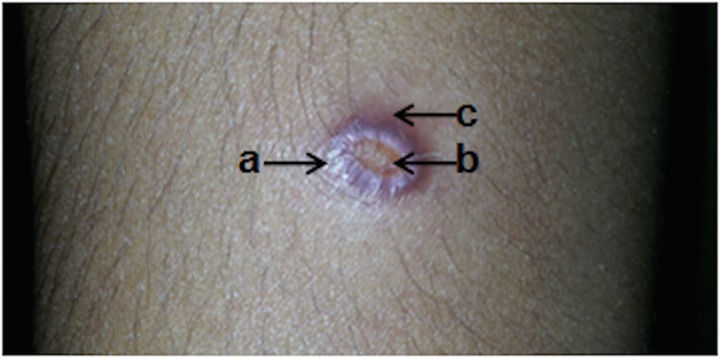
An ulcerating skin nodule surrounded by an erythromatous reaction in the upper limb of the CL patient. Commonest presentation (*n* = 80/250, 32%) of cutaneous leishmaniasis patients included in the study. (a) Nodular area; (b) central ulceration: (c) surrounding skin with erythromatous reaction.

Lesion types seen were erythromatous papules (*n* = 34), nodules (*n* = 58), plaques (*n* = 39), ulcers (*n* = 47), ulcerating nodules (*n* = 80) and other variations (*n* = 11). A number of lesions varied from 1 to 3, with the majority (*n* = 242, 96·8%) having single lesions. The size of lesions ranged from 1 to 5 cm (the median diameter of a lesion was 1·5 cm). The most common clinical feature was a single lesion of an ulcerating nodule surrounded by a notable erythromatous reaction (*n* = 80, 32%) (Fig. 1). Lesions were mostly observed on upper limbs and the face (upper limbs *n* = 106, 39·4%, face *n* = 82, 30·5%) of otherwise healthy individuals. The time between the appearance of lesion and diagnosis varied from 1 month to 1 year, with a median of 6 months.

None of the patients included in the study showed any symptoms or signs of pallor/jaundice, loss of appetite, loss of weight, splenomegaly or liver enlargements at the onset of the study. Haematological investigations done at the time of recruitment for the study to assess RBC count, WBC count, platelet count, haemoglobin content, packed cell volume and albumin:globulin ratio were within the reference ranges (RBS: 3·9–5·6 × 10^12^ L^−1^; WBC: 4–11 × 10^9^ L^−1^; platelet count: 150–400 × 10^9^ L^−1^; Hb: 12·65–15·65 g dL^−1^; PCV: 38–45·5 L L^−1^; A/G: 0·8–2·3). Serological assay for anti-*L. donovani* antibodies (rk39 dipstick assay) was negative in all patients. All diagnosed patients included in this study were treated with weekly 1–2 mL IL-SSG (Pentostam, GSK, 100 mg mL^−1^) injections until cure. The lesions healed in 76·4% of the patients by the end of ten standard doses of IL-SSG and the remaining patients (*n* = 59, 23·6%) were cured following extra four to nine doses of IL-SSG.

### Patient follow-up

Haematological investigations remained within the normal range in most part with no significant deviations detected during the 4-year period. There were no signs of regional lymph-adenopathy, visceral involvement such as pallor/jaundice, loss of appetite, loss of weight, splenomegaly or hepatomegaly during this follow-up period. Similarly, there was no evidence for the presence of anti-K39 antibodies in any of these patients as evidenced by negative rk39 assays throughout the follow-up period. Recurrence of skin lesions was observed in 3·6% of patients (*n* = 9) at 18 and 24 months from the initial diagnosis. The newly recurred lesions were found either on the exact same spot of the initial lesion or in the vicinity (within 2 cm of the initial lesion). Tissue scrapings from healed lesions (following completion of IL-SSG treatment) were tested for the presence of amastigotes through microscopy and culture. However, amastigotes were not found in any of the samples, even those from recurrent lesions. Patients with recurrent lesions were referred back to the Dermatologists and were treated successfully with either intra-lesional SSG injections (IL-SSG) or direct spray of liquid nitrogen (cryotherapy) (WHO Expert Committee, 2010).

### *In vivo* virulence of *Leishmania* parasites in animal models

**Fig. 2.**
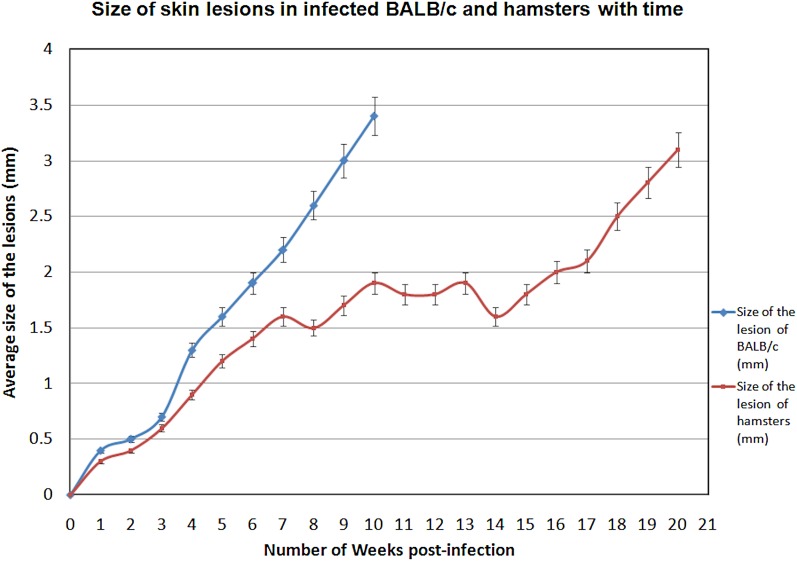
Size of skin lesions in infected BALB/c and hamsters against time. Animals were infected with *L. donovani*-SL promastigotes intra-dermally in the ear pinna and lesion appearance and progression were monitored weekly for a period of 10 weeks for BALB/c and 20 weeks for hamsters. Data shown indicate the mean lesion size ± s.d. in each experimental group.

Parasite burden in spleen and LNs of infected animals were analysed. Six infected BALB/c mice (B-ID-SL) euthanized at 5 weeks (40%) and 16 infected BALB/c mice euthanized at 10 weeks (76·2%) showed cutaneous lesions at the site of inoculation in the ear pinna. However, there were no signs of systemic infection in any of these animals. Only 11/25 (44%) hamsters injected intra-dermally (H-ID-SL), showed cutaneous lesions at the site of parasite inoculation and no signs of systemic infection was evident. There was no evidence for infection of the spleen, liver and draining LNs in any of the infected animals. The skin lesions seen in both BALB/c mice and hamsters were of the nodular type, which increased in size gradually until sacrifice (Fig. 2). There was no significant body weight gain/loss in any of these animals at the end of the study period (Table 1).

**Table 1.** The weight of animals and their organs

Animal		Mean weight (g)			Mean % increase in body weight^a^	*P* value
		Body	Spleen	Liver		
BALB/c mice	Infected *n* = 14	27·59 ± 0·63 (10·74 ± 0·36)	0·09 ± 0·005	1·19 ± 0·05	157·00	0·105
	Control *n* = 8	27·74 ± 0·51 (11·02 ± 0·39)	0·11 ± 0·006	1·16 ± 0·12	152·00	
Syrian golden hamsters	Infected *n* = 11	147·31 ± 0·56 (42·25 ± 0·48)	1·78 ± 0·14	3·08 ± 0·008	249·00	0·064
	Control *n* = 5	160·58 ± 0·38 (37·02 ± 0·19	0·67 ± 0·14	1·30 ± 0·12	334·00	

The average body weight, weight of liver and spleen at the time of euthanization in each group of animals injected with parasite isolates *via* intra-dermal route is indicated in the table. Values are mean ± 1 s.d. from five separate sets of observations. The original body weight of animals prior to injection is given within parenthesis.

a No statistical differences were observed in the body weight or the organ weight of the test animals when compared with the control group. Increased body weight at the time of euthanization was expressed as a percentage of weight at onset.

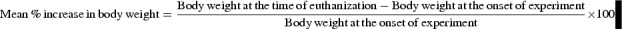

**Fig. 3.**
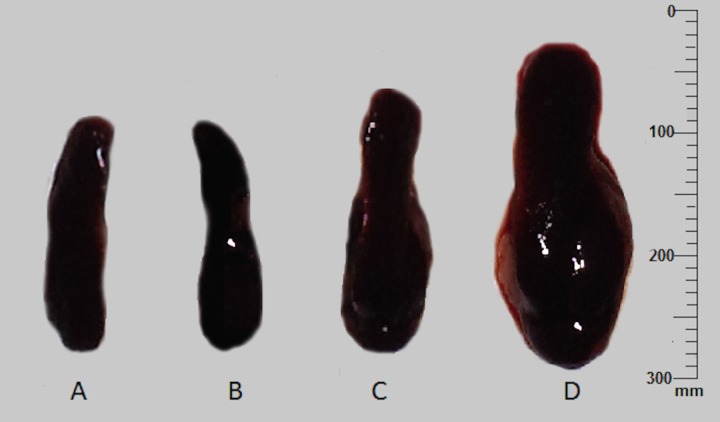
Spleen enlargement of the BALB/c mice at 10 weeks post-infection. Spleen from mice administered with normal saline (A), with *L. donovani*-SL *via* intra-dermal route (B), with *L. donovani*-SL *via* intra-venous route (C) and with *L. donovani-*1S, *via* intra-dermal route (D).

In terms of induced skin infections, the percentage of infection and parasite burden in the ear lesion were comparable in both animal models tested. However, in terms of systemic infections, high parasite burden in the spleen was observed in intra-venously infected BALB/c mice (ranged 65–140 LDU), than the intra-cardially infected hamsters (ranged 25–48 LDU). Interestingly, all BALB/c mice similarly infected ID with *L. donovani*-1S, showed noticeable enlargement of the spleen with high parasite burden both in the spleen (average = 348 ± 6·9 LDU) and the LNs (average = 106 ± 2·1 LDU) (Fig. 3).

### *In vitro* cytokine stimulation of splenocytes and LN cells of infected BALB/c mice

**Fig. 4.**
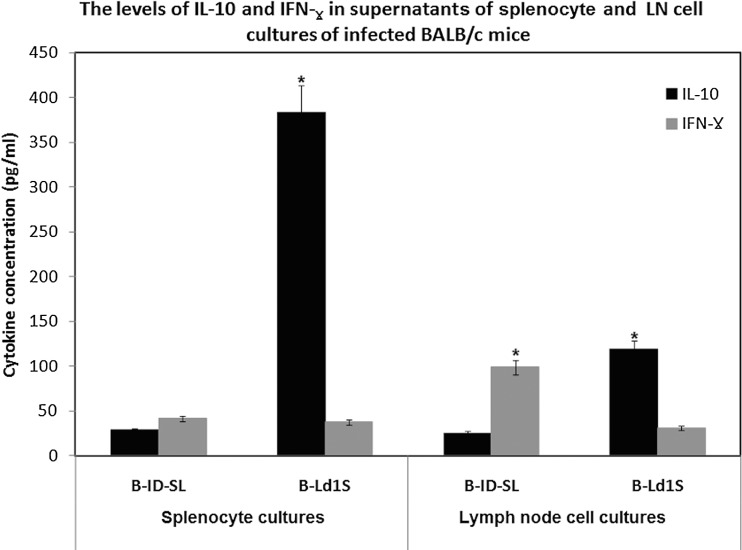
The level of cytokines measured in splenocytes and lymph node (LN) cell culture supernatants of BALB/c mice, euthanized at 10 weeks post-infection, followed by *in vitro* SLA stimulation. B-ID-SL: mice infected intra-dermally with *L. donovani*-SL, B-Ld1S: mice infected with *L. donovani*-1S. The level of IL-10 and IFN-γ measured in splenocyte and LN cell culture suspensions following stimulation with SLA. Splenocyte and LN cell culture supernatants were collected after 72 h and IL-10 and IFN-γ concentrations determined by ELISA. *P < 0·05. SLA, Soluble *Leishmania* antigen.

There was a marked difference in IFN-γ: IL-10 ratio in parasite-stimulated culture supernatants of splenocytes derived from mice infected with either *L. donovani*-SL (1.5: 1; 41·37 ± 1·25 pg mL^−1^: 28·47 ± 0·85 pg mL^−1^) or *L. donovani*-1S (1 : 10; 37·46 ± 1·21 pg mL^−1^: 383·37 ± 7·67 pg mL^−1^, *P* > 0·05). Similar trend was also seen with LN cell cultures with IFN-γ: IL-10 ratios being 4 : 1 (99·05 ± 2·97 pg mL^−1^: 25·10 ± 0·75 pg mL^−1^) for *L. donovani*-SL and 1 : 4 (119·15 ± 2·38 pg mL^−1^: 30·80 ± 0·92 pg mL^−1^, *P* < 0·05) for *L. donovani*-1S derived cells, respectively (Fig. 4).

### *In vitro* infection of human macrophages

**Fig. 5.**
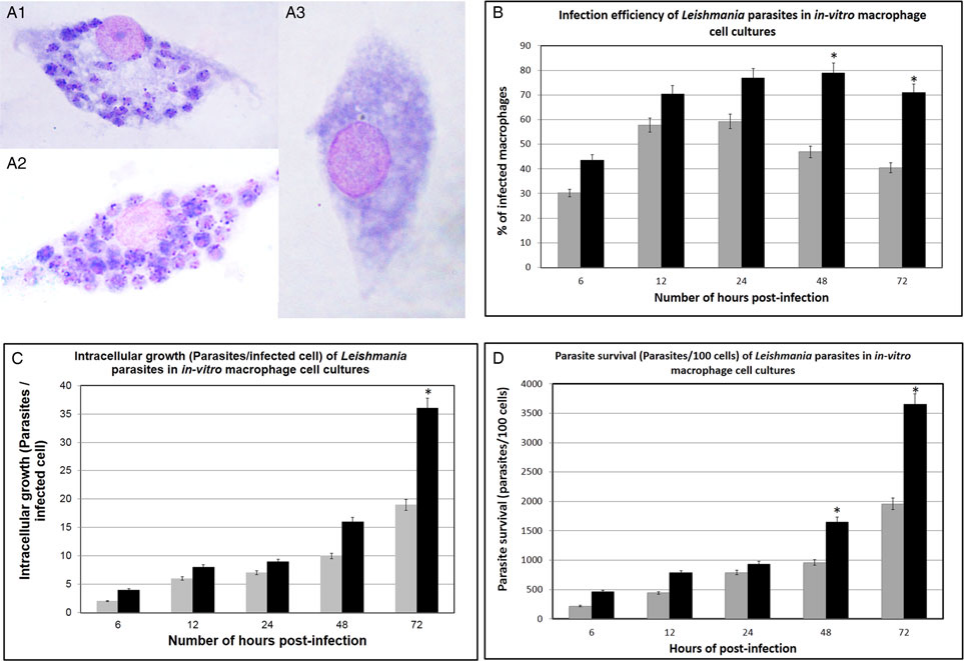
*In vitro* macrophage infections with *L. donovani*-SL and *L. donovani*-1S. Images of macrophages after 48 h post-infection with (A1) *L. donovani-*SL parasites; (A2) *L. donovani*-1S; (A3) Un-infected macrophages (negative control). Percentage of infected macrophages (B); Rate of intracellular growth (number of Parasites/infected cell) (C); Intracellular parasite survival (Parasites/100 cells) (D) were measured at 6, 12, 24, 48 and 72 h following parasite inoculation in the macrophages *ex vivo*. *P < 0·05.

*Leishmania donovani-*1S and *L. donovani-*SL infected macrophages, along with the uninfected cells are shown in Figs 5A1–A3. When compared with *L. donovani*-1S-infected mice, infection efficiency of the local parasite was lower with peak levels (59·25%) reached at 24 h post-infection, followed by a subsequent reduction. In contrast, for *L. donovani-*1S, peak infection efficiency (78·95%) was observed at 48 h post-infection (Fig. 5B).

*In vitro* intracellular growth of *L. donovani-*1S parasites was almost double that of the *L. donovani*-SL at 72 h (*P* < 0·05) (Fig. 5C). Parasite survival increased with time following infection in both the species (*L. donovani*-1S and *L. donovani-*SL). The survival rate of *L. donovani-*1S was as twice as that of the local parasites, at 48 and 72 h post-infection (*P* < 0·05) (Fig. 5D).

## DISCUSSION

The visceralizing potential of Sri Lanka *L. donovani* parasites was investigated through long-term follow-up of CL patients and with the use of both *in vivo* and *in vitro* models of experimental infection. Furthermore, successful attempts were made to establish an animal model, which was subsequently used for comparative virulence studies between *L. donovani*-SL and *L. donovani*-1S, a known visceralizing strain of *Leishmania*. The absence of any systemic infection in apparently cured CL patients during the follow-up period of 4 years and evidence gathered through *in vitro* experiments and animal studies, support the dermotropic nature of *L. donovani* in Sri Lanka.

This study documents the first long-term patient follow-up to investigate leishmaniasis disease sequelae in Sri Lanka. The absence of signs and symptoms of visceralization in these patients with currently available markers confirms the essential dermotropic nature of *L. donovani* in the country. Though remote, a possibility exists that the available biomarkers, as at present, including rk39 dipstick assay, failed to detect the progression of disease or early systemic infections in this study due to limitations in sensitivity and specificity, a view however, that remains speculative. Recurrence of CL observed among a few patients (*n* = 9) indicates the importance of follow-ups and raising awareness about this disease among the patients. The recurrence of lesions might be due to the persistence of residual parasites in the skin of apparently cured CL patients, even after antimonial therapy. However, this study failed to demonstrate the presence of residual parasites after the completion of antimony therapy that might be due to inadequate sensitivity of microscopy and culture that were used as evidence of infection; a possibility that could be further investigated using more sensitive tools for parasite detection.

The dermotropic properties of viscerotropic *L. donovani* parasites have been similarly observed in countries such as Kenya, Yemen and Ethiopia (Mebrahtu *et al.* 1993; Pratlongl *et al.* 1995; Gelanew *et al.* 2011). Furthermore, CL due to *L. donovani*/*L. infantum* hybrid has been reported from Northern Cyprus and Turkey (Antoniou *et al.* 2008; Svobodová *et al.* 2009), while CL causing *L. infantum* has been found in Iran, Israel and France (Giudice *et al.* 1998). These studies have suggested the genetic divergence of the usually viscerotropic parasite species with CL as the resultant disease outcome.

A major observation of this study was that intra-dermal infection of mice with live *L. donovani*-SL isolates did not lead to visceralization or systemic spread of infection, whereas intra-venous infection of mice or intra-cardial infection of golden hamsters using the same parasite isolates (data not shown) did. This demonstrates the intrinsic capacity of Sri Lankan *L. donovani* to visceralize. Non-visceralization of *L. donovani*-SL introduced *via* the intra-dermal route, may be due to the in-situ-immune responses evoked by *L. donovani*-SL, which has been demonstrated by our group (Manamperi *et al.* 2017). In contrast, intra-venous or intra-cardial infection is likely to have by-passed the *in situ* host immunity. Therefore, it could be suggested that *L. donovani*-SL parasites possess the ability to visceralize in the event that it could successfully by-pass the local tissue immune reactions at the site of inoculation. Vector sand flies also play a vital role in determining the disease outcome. During the natural process of infection, sandflies inject 10^2^–10^4^ promastigotes into the skin to produce a small wound or for visceralization (Sacks and Melby, 2001). As this study shows, the parasites that by pass the local tissue response and directly enter the circulation, may indeed establish systemic infection, if they are able to overcome host defence mechanisms.

The use of a mouse model limits the ability to replicate human skin infection due to the differences in the structure of skin between mice and humans. Therefore, caution needs to be exercised in relating the outcome of ID infection of mice to humans due to differences in dermal immunity between the two (Loeuillet *et al.* 2016). In this study, all animals infected ID did not show skin lesions, possibly due to many reasons including biological variations between animals. Though remote, inconsistencies in techniques used also may have contributed to such variations. The disease outcome is significantly influenced by the interactions between macrophages and parasites (Sariaslani and Gadd, 2013). Lower infection efficiency, intra-cellular growth and parasite survival of *L. donovani-*SL, in comparison with *L. donovani*-1S in the *in vitro* experiments might also contribute to the ‘atypical’ outcome of *L. donovani* infection apparent as a cutaneous disease in Sri Lanka. However, the exact mechanism by which the visceralizing strains by pass the local tissue defence mechanisms during natural infection remains unclear.

Significant levels of IFN-γ (and low levels of IL-10) seen in parasite antigen-stimulated LN cell cultures from CL-acquired animals (B-ID-SL), might support the role of IFN-γ in limiting the localization of parasites within tissues in the vicinity of the cutaneous lesions, without the subsequent systemic spread. This theory is in fact supported by our recent findings through cytokine expression studies done on lesion tissues of CL patients that demonstrated a prominent Th1-biased local immune response (Manamperi *et al.* 2017). Furthermore, splenocytes and LN cells of animals with systemic infections of *L. donovani*-SL, demonstrated the ability to produce elevated levels of IL-10 but only low levels of IFN-γ, following parasite antigen stimulation (data not shown). This might point towards a Th2 bias associated with systemic involvement and progressive disease (Gupta *et al.* 2013).

Overall, the adaptation of parasites to the vector or the host reservoir in diverse epidemiological settings may have forced it to undergo genetic changes, resulting in its dermotropic nature. On the other hand, the nature of the host immune response at the site of parasite inoculation also may influence the disease sequelae. A complete understanding of the immunopathological basis of disease manifestation of CL-inducing *L. donovani* in Sri Lanka, however, would require further investigation.
